# Two-Photon Absorption and Dynamics of Excited States in Bromochalcone Derivatives

**DOI:** 10.1021/acs.jpca.5c02748

**Published:** 2025-09-30

**Authors:** Nathan B. Marucci, João V. P. Valverde, Gabriel de O. Campos, Eli S. A. Ducas, Pablo J. Gonçalves, Leonardo De Boni, Cleber R. Mendonça

**Affiliations:** † Photonics Group, São Carlos Institute of Physics, 28133 University of São Paulo, CP 369, São Carlos, São Paulo 13566-590, Brazil; ‡ Institute of Chemistry, 67824 Federal University of Goias, Goiânia, Goias 74690-900, Brazil; § Superintendence of Construction Management, Water Treatment and Sanitation Company of Goias, Goiânia, Goias 74805-100, Brazil; ∥ Institute of Physics, Federal University of Goias, Goiania, Goias 74690-900, Brazil; ⊥ Center of Excellence in Hydrogen and Sustainable Energy Technologies (CEHTES), Goiania, Goias 74690-631, Brazil

## Abstract

Two-photon absorption (2PA) in organic compounds has gained significant interest due to its applications in nonlinear optics, including two-photon fluorescence microscopy, photodynamic therapy, and laser microfabrication. This study investigates the two-photon absorption cross section (2PACS) and the excited-state dynamics of 4′-bromochalcone derivatives using a combination of experimental and computational approaches. Quantum chemical calculations employing density functional theory (DFT) and time-dependent DFT (TD-DFT) were performed to analyze the electronic properties of the molecules. Experimental characterization involved linear optical measurements (UV–vis absorption and fluorescence spectroscopy), femtosecond transient absorption spectroscopy (TAS), and open-aperture (AO) Z-scan measurements to determine the degenerate 2PACS (D-2PACS). Our results reveal that resonant donor substituents, such as the dimethylamino group, enhance nonlinear absorption, especially in the lower-energy band. Furthermore, TAS revealed an intriguing dynamic associated with the formation of a twisted intramolecular charge transfer (TICT) state, which was corroborated by anisotropy and solvatochromism measurements as well as by computational simulations. These findings provide insights into the relationship between the molecular structure and nonlinear optical properties, contributing to the development of optimized materials for photonic applications.

## Graphical abstract

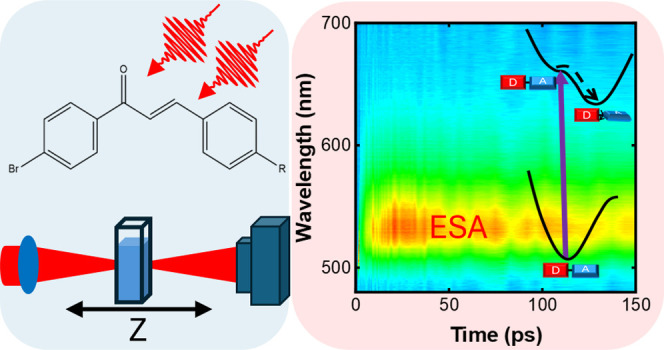

## Introduction

The interaction between light and matter has long been a subject of scientific interest. Among the various related phenomena, nonlinear optics^1^ stands out as a particularly intriguing area, owing to its broad range of potential applications including optoelectronics, frequency conversion,^2,3^ optical power limiting,^4,5^ all-optical switching,^6^ biological imaging,^7–9^ 3D microfabrication,^10,11^ photodynamic therapy,^12^ and so on. Designing and developing materials for these applications remain major challenge. Despite the wide variety of nonlinear optical (NLO) materials reported in the literature, the pursuit of efficient, cost-effective, and synthetically accessible compounds with strong nonlinear optical responses is still ongoing. Within this scope, chalcone derivatives have gained increasing attention in recent decades as a promising molecular system for NLO applications.^13–22^

Chalcones are organic compounds within the flavonoid group that serve as key precursors in the biosynthesis of various natural products. These molecules are found in many plants and exhibit a range of biological and pharmacological activities, such as antioxidant, antitumoral, antibacterial, antifungal, etc.^23–27^ Structurally, chalcones consist of two aromatic rings linked by a α,β-unsaturated ketone. Their straightforward synthesis facilitates the molecular engineering of novel compounds by incorporation of different substituents into the aromatic rings. While chalcones have been extensively studied for biological applications, their potential in the field of NLO materials continues to attract research interest. Literature reports indicate that electron delocalization via the π-conjugated system combined with the presence of electron-donating and electron-withdrawing groups can significantly enhance their nonlinearities.^28^

In this context, the present work aims to investigate the linear and NLO properties—specifically two-photon absorption (2PA)—of a series of 4′-bromochalcone derivatives, focusing on how different substituents on the aromatic ring affect their behavior.

Figure 1 presents the molecular structures of the studied chalcones. All samples share a bromine atom in the *para* position of the aromatic ring adjacent to the carbonyl group (ring A) and differ by the nature of the substituent in the *para* position of the other ring (ring B). This molecular design strategy preserves the structure of ring A to minimize variability while allowing systematic modifications on ring B, enabling a consistent comparison of the effects of different functional groups.

**Figure 1:**
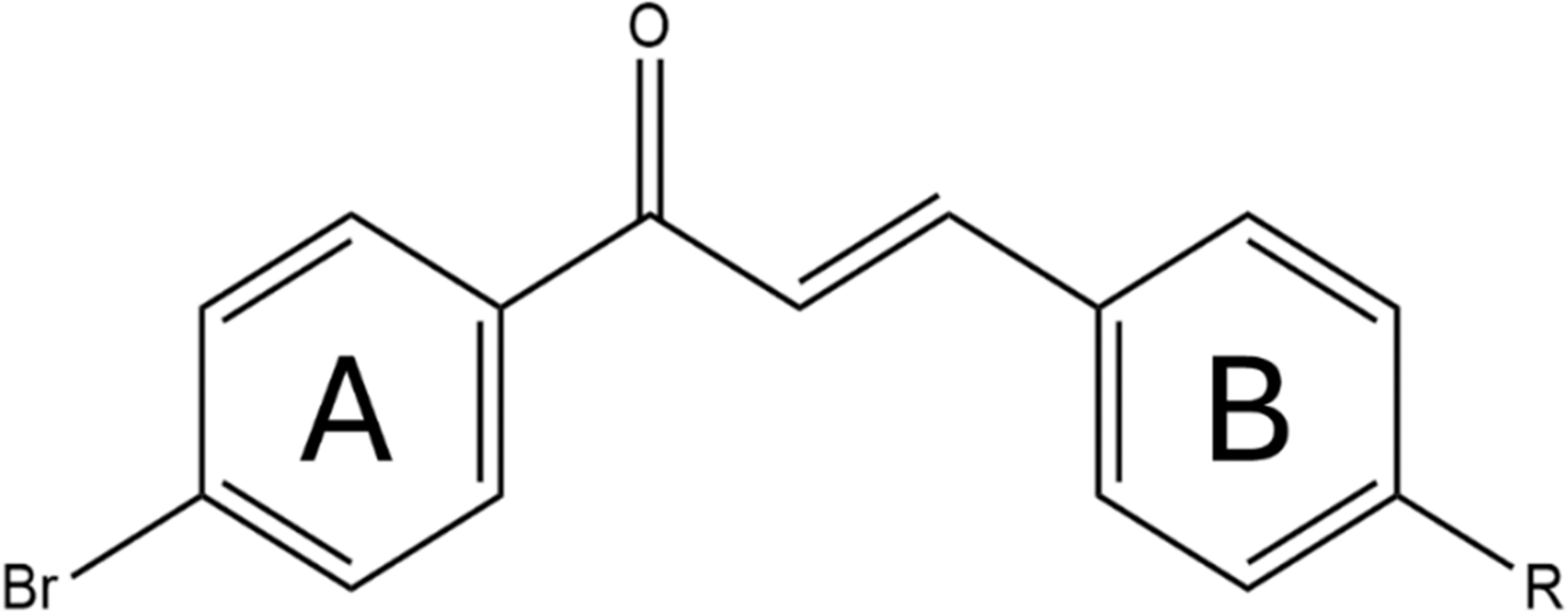
Molecular structure of 4′-bromochalcone derivatives. R represents the functionalized side group in the main backbone.

Experimental characterization involved linear optical techniques (UV–vis absorption and fluorescence spectroscopy), femtosecond transient absorption spectroscopy (TAS), and open-angle (OA) Z-scan measurements. Additionally, to support our experimental findings, we carried out quantum chemical calculations based on the density functional theory (DFT) framework and its time-dependent variant (TD-DFT).

## Experimental Section

The studied compounds are synthesized by the Claisen–Schmidt reaction between 4-bromoacetophenone (ring A) and others aromatic aldehydes (ring B) in the same method described in reference.^29^

Table 1 shows the different substituents placed as side group R displayed in Figure 1. Accordingly, the ten compounds studied in this work are (*E*)-1-(4-bromophenyl)-3-phenylprop-2-en-1-one (**A1**); (*E*)-1-(4-bromophenyl)-3-(*p*-tolyl)­prop-2-en-1-one (**A2**); (*E*)-1-(4-bromophenyl)-3-(4-ethylphenyl)­prop-2-en-1-one (**A3**); (*E*)-1-(4-bromophenyl)-3-(4-methoxyphenyl)­prop-2-en-1-one (**A4**); (*E*)-1-(4-bromophenyl)-3-(4-ethoxyphenyl)­prop-2-en-1-one (**A5**); (*E*)-1-(4-bromophenyl)-3-(4-(dimethylamino)­phenyl)­prop-2-en-1-one (**A6**); (*E*)-1-(4-bromophenyl)-3-(4-fluorophenyl)­prop-2-en-1-one (**A7**); (*E*)-1-(4-bromophenyl)-3-(4-chlorophenyl)­prop-2-en-1-one (**A8**); (*E*)-1,3-bis­(4-bromophenyl)­prop-2-en-1-one (**A9**); and (*E*)-1-(4-bromophenyl)-3-(4-nitrophenyl)­prop-2-en-1-one (**A10**).

**Table 1:** Substituent Groups at the *Para* Position of 4′-Bromochalcone Molecules

	**A1**	**A2**	**A3**	**A4**	**A5**	**A6**	**A7**	**A8**	**A9**	**A10**
R	H	–CH_3_	–CH_2_CH_3_	–OCH_3_	–OCH_2_CH_3_	–N(CH_3_)_2_	F	Cl	Br	–NO_2_

We prepared solutions with 10^–2^ M concentrations in dimethyl sulfoxide (DMSO) and used them for the 2PA experiments. Subsequently, we diluted 100 μL of these solutions in 10 mL of DMSO to produce diluted solutions (∼10^–4^ M concentrations), which we used for linear optical measurements.

### Linear Measurements

We measured the UV–vis absorption and fluorescence emission spectra at room temperature by using Shimadzu UV-1800 and Hitachi F-7000 spectrophotometers, respectively. For absorption measurements, we used a 2 mm quartz cuvette, and for fluorescence measurements, we used a 1 cm cuvette. We can estimate the transition dipole moment between the ground state and the excited state using the following equation obtained from the time-dependent perturbation theory^30^

1 $$\left\langle \sigma_{0f}^{\left(1\right)}\right\rangle =\frac{\pi \omega }{3n\epsilon_{0}c}\frac{{\left|\mu_{0f}\right|}^{2}}{\hbar }\rho_{f}\left(\omega_{0f}=\omega \right)$$

### Fluorescent Properties

To determine the fluorescence quantum yield (ϕ_fl_) of the 4′-bromochalcone derivatives, we employed eq 2.^31^ We used a chalcone molecule reported in the literature^29^ as a reference sample (ϕ_fl_ = 71% in DMSO) and measured all samples’ emission and absorption spectra under the same experimental condition.

2 $$\phi_{fl}=\phi_{fl_{R}}\times \frac{\mathrm{Int}}{Int_{R}}\times \frac{1-10^{-Abs_{R}}}{1-10^{-Abs}}\times \frac{n^{2}}{n_{R}^{2}}$$

In eq 2, the subscript R refers to the reference sample, Int is the area under the fluorescence curve, Abs is the absorbance, and *n* is the refractive index.

To characterize the fluorescence lifetime τ_fl_, we used a 2 mm cuvette. We excited the samples using a regenerative amplified Yb:KGW femtosecond laser system (PHAROS PH1 model, Light Conversion), operating at 343 nm (third harmonic of 1030 nm) with a pulse duration of approximately 220 fs and a repetition rate of 300 Hz. A converging lens focused the laser beam, placing the sample slightly beyond the focal point. We collected the fluorescence perpendicular to the excitation, using an optical fiber directed to a photodetector.

We measured the steady-state anisotropy spectrum, ⟨*r*⟩, using a fluorimeter adapted with two polarizers: one for the excitation light polarization and the other one for the emission polarization. We measured the emission intensity as a function of the excitation wavelength for four polarization combinations: VV, VH, HV, and HH, where the first letter denotes the polarization of the excitation (vertical or horizontal) and the second refers to the emission. Then, we determined ⟨*r*⟩ using^32,33^

3 $$\left\langle r\right\rangle =\frac{I_{VV}-GI_{VH}}{I_{VV}+2GI_{VH}}$$

with $G=\frac{I_{HV}}{I_{HH}}$ being an experimental apparatus correction factor.

We repeated the ⟨*r*⟩ measurements after adding small amounts of glycerol to modify the viscosity and plot the Perrin equation.^32,34,35^ To determine the viscosity of the DMSO and glycerol mixture, we used the Arrhenius equation^36^

4 $$\ln\eta =x_{1}\ln\eta_{1}+x_{2}\ln\eta_{2}$$

where *x*_*i*_ is the molar fraction of the *i*th component of the mixture.

To measure solvatochromism, we dissolved the sample in solvents with different polarities and measured the linear absorption and emission spectra to plot the Lippert–Mataga equation (eq 5) and determine the Δ**μ**_01_ ≡ **μ**_11_ – **μ**_00_ value. The Onsager radius, *a*, was determined by using the anisotropy measurements as described in reference.^37^

5 $${\mathrm{\nu ̅}}_{A}-{\mathrm{\nu ̅}}_{F}=\frac{2{\left|\Delta \mu_{01}\right|}^{2}}{a^{3}hc}\left[\frac{\epsilon -1}{2\epsilon +1}-\frac{n^{2}-1}{2n^{2}+1}\right]+cte$$

### Transient Absorption Measurements

Femtosecond transient absorption spectroscopy (TAS) measurements^38^ were performed using a custom-built pump–probe setup to investigate excited-state dynamics.^39^ The same laser system described in the previous section was used for this purpose. We used half of the laser power to pump an optical parametric amplifier (OPA) (Orpheus model, Light Conversion), generating a tunable pump pulse at 430 nm for molecule **A6** and 340 nm for the others. The other portion of the laser was used to generate a white-light supercontinuum (480–700 nm) probe pulse by focusing the beam onto a sapphire crystal. We carried out the measurements at the magic angle and used Glotaran^40^ software for the global analysis of the experimental data.

### Nonlinear Optical Measurements

We determine the degenerate 2PACS (D-2PACS) spectra of the 4′-bromochalcone derivatives using the open-aperture (OA) Z-scan technique.^41^ We used the same laser system and the previously described OPA operating at a repetition rate of 750 Hz. The OPA enabled us to tune the excitation wavelength from the visible to near-infrared. In this case, we performed the measurements from 550 nm with increments of 10 nm up to approximately 700 nm depending on the molecule. For more information about the experimental setup, see the Supporting Information.

### Quantum Chemical Calculations

Quantum chemical calculations were carried out using Gaussian 09 software^42^ to analyze the electronic and structural properties of the compounds. We performed geometry optimization using the DFT level, and based on the optimized structures, we calculated electronic transition characteristics employing the TD-DFT variant. We used the PBE1PBE functional^43^ and the 6-311++g­(d,p) basis set^44^ in these calculations. To account for the effect of the DMSO, we used the polarizable continuum model (PCM) with the integral equation formalism (IEF-PCM).^45,46^ We used the Multiwfn software^47^ to calculate the natural transition orbitals (NTOs) of the first electronic transitions. We computed NTOs for all samples except samples **A4**, **A5**, and **A6**, which exhibit high contribution coefficients. To plot the simulated absorption spectra, we used a fwhm of 0.33 eV for all transitions.

## Results and Discussion

### One- and Two-Photon Absorption Spectra

Figure 2 presents the degenerate 2PA spectra (red circles) overlaid with the corresponding 1PA spectra (black lines). It is worth noting that the addition of substituents in the *para* position of the B ring shifted the absorption spectra toward longer wavelengths compared to the reference sample **A1**, whose peak is at 318 nm (see Table 2). Notably, sample **A6** exhibited the highest bathochromic shift with peak absorption at 432 nm. Close behind, molecules **A4** and **A5** had identical peak absorption at 348 nm.

**Figure 2:**
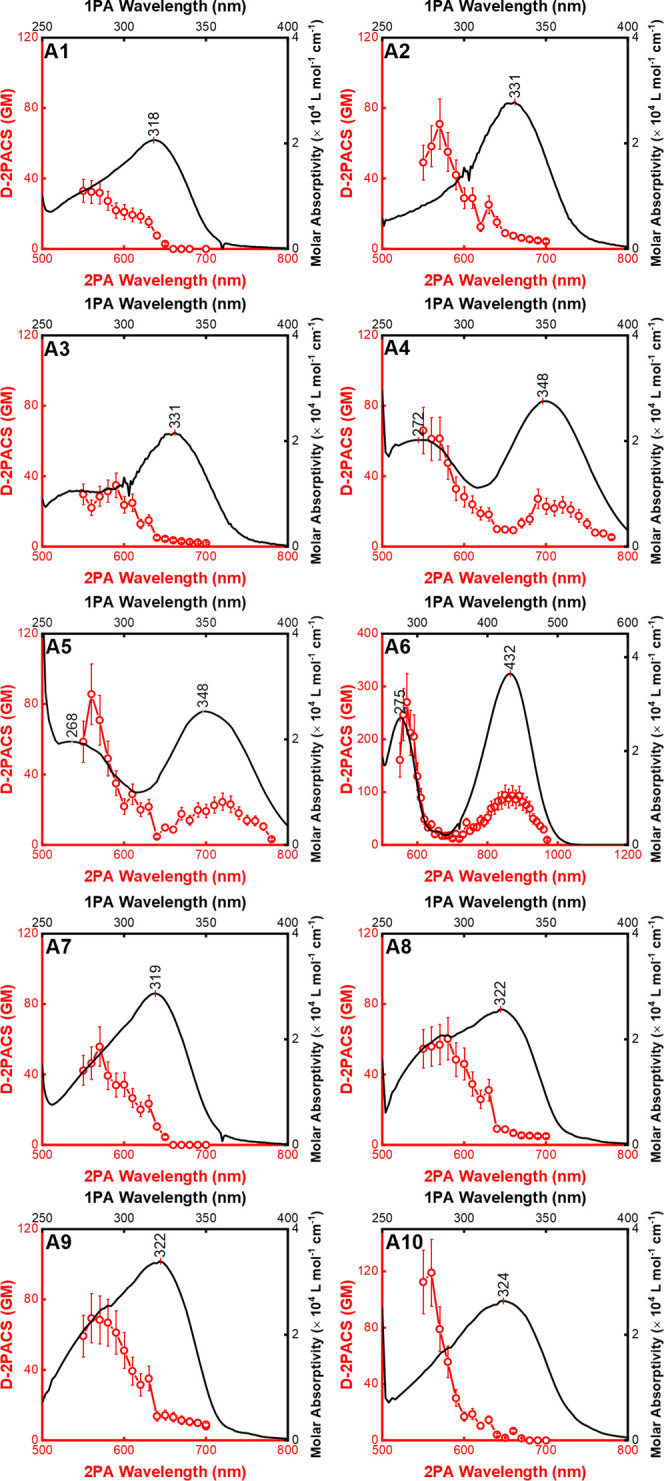
Degenerate two-photon absorption cross section (D-2PACS) spectra (red circles: left and bottom axes) and one-photon absorption spectra (black lines: right and top axes) of 4′-bromochalcone derivatives in DMSO.

**Table 2:** Maximum Absorption Wavelength and Transition Dipole Moment from the Ground State to the First Excited State

	**A1**	**A2**	**A3**	**A4**	**A5**	**A6**	**A7**	**A8**	**A9**	**A10**
λ_max_ (nm)	318	331	331	348	348	432	319	322	322	324
μ_01_ (D)	5.7	7.7	6.3	8.2	7.7	9.6	6.7	6.6	7.6	6.9

Table 2 displays the maximum one-photon absorption wavelengths (λ_max_) along with the μ_01_ values, which were obtained by decomposing the experimental linear absorption curves into Gaussian functions (see Figure S4 in the Supporting Information for decomposed curves).

The magnitude of the lower-energy band (10^4^ L mol^–1^ cm^–1^) suggests that it involves π → π* transitions, as shown in Figure 3 through the computational simulations for molecules **A1** and **A6**, with their corresponding theoretical one-photon absorption spectra. For information on other molecules, see Figures S8–S11 in the Supporting Information. As we can see, the low-energy transition for all samples—except **A4**, **A5**, and **A6**—is of the n → π* type, involving the nonbonding electron of the carbonyl group, resulting in low oscillator strength. On the other hand, in these samples, the S_2_ state is a ππ* state with a high oscillator strength. Molecules **A4**, **A5**, and **A6** exhibit only one electronic transition in the lower-energy band involving a ππ* state.

**Figure 3:**
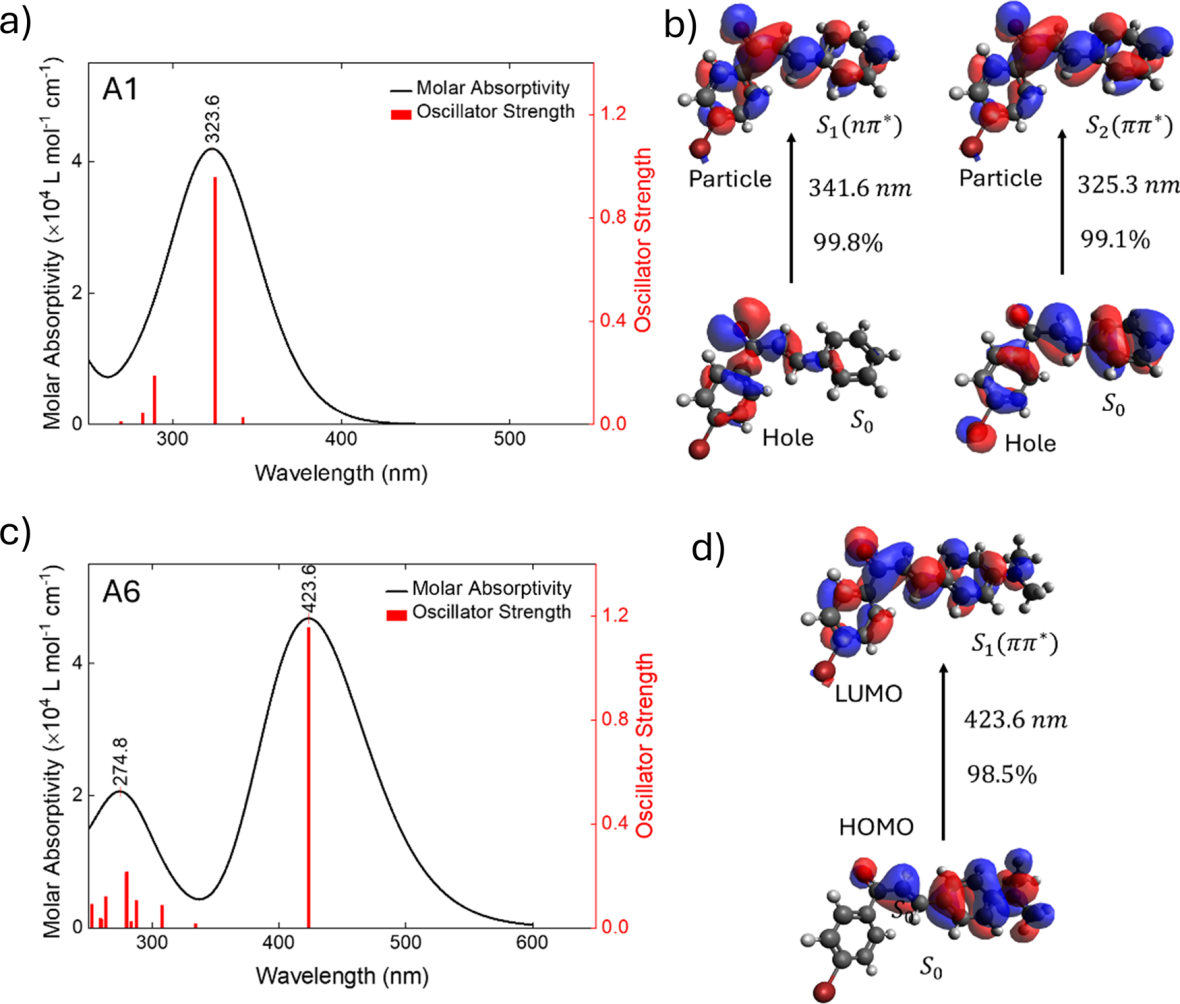
Time-dependent simulation results: (a) one-photon absorption spectra and (b) NTOs for the first and second low-energy transitions for molecule **A1**, (c) one-photon absorption spectra, and (d) frontier orbitals for molecule **A6**.

From the 2PA spectra, we observe that the most energetic states are more accessible via two-photon absorption, with most of the molecules, except for **A4**, **A5**, and **A6**—exhibiting negligible values in the lower-energy band. From geometry optimization (Figure 4), we observed that the samples are nonplanar, likely due to a steric hindrance between hydrogen atoms, which causes twisting around the carbonyl group region (Figure 4a). This disruption of conjugation may explain the low 2PACS obtained for chalcone, in general. However, when examining the exceptions—**A4**, **A5**, and **A6** (Figure 4b), with the first two exhibiting a D-2PACS value of approximately 23 GM in the lower-energy band and **A6** reaching around 90 GM—we see that the oxygen and nitrogen of the substituent groups show a trigonal planar geometry, i.e., sp^2^ hybridization with a lone pair of electrons in a p orbital perpendicular to the other three hybridized orbitals of these atoms (see Figure 4c for nitrogen), allowing the overlap of the p orbital with the p orbitals of the carbons in the aromatic ring, donating these electrons to the conjugated system. The extension of the π-conjugated system in these samples also explains their greater bathochromic shift.^48^ Thus, although oxygen and nitrogen are more electronegative than carbon and have an electron-withdrawing inductive effect, they present mesomeric (resonant) donor characteristics (Figure 4d). Although halogens also have p orbitals with nonbonding electrons, the overlap of these orbitals with the aromatic ring decreases as the atomic size increases. Thus, for these samples as well as for **A2** and **A3**, the inductive effects prevail, with no contribution to nonlinear absorption. Although the nitro group (–NO_2_) in **A10** has resonant nonbonding electrons, this functional group exhibits a very strong electron-withdrawing mesomeric (and inductive) character, pulling the electron density toward it, “deactivating” the aromatic ring and decreasing the 2PA.^49^

**Figure 4:**
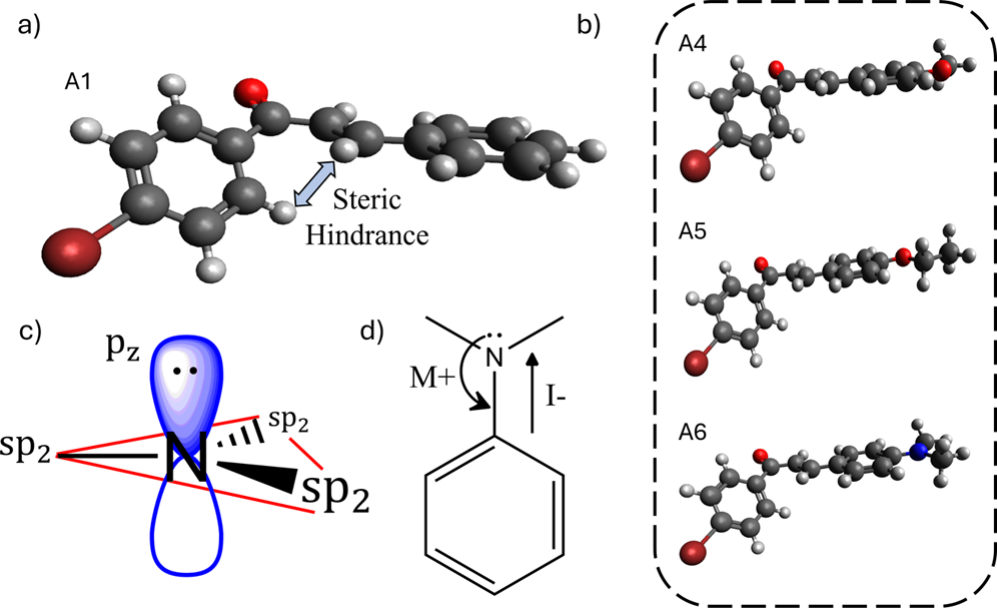
(a) Optimized structure of molecule **A1** showing steric effects between hydrogen atoms, (b) optimized structures of molecules **A4**, **A5**, and **A6**, (c) sp^2^ nitrogen hybridization, and (d) inductive and mesomeric (resonant) effects for the amine group.

### Fluorescent Properties

Following the fluorescence measurements, **A6** is the only one with significant fluorescence, showing a quantum yield of ca 14%. Figure 5a presents the absorption and emission spectra of this molecule, revealing a large Stokes shift of 126 nm (0.65 eV). Figure 5b presents the emission spectrum of this sample for different excitation wavelengths, demonstrating that Kasha’s rule^50^ is obeyed.

**Figure 5:**
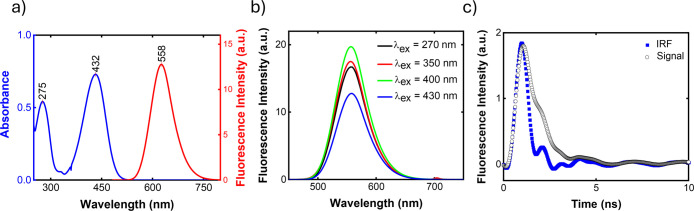
Fluorescence results: (a) absorption and emission spectra, (b) fluorescence spectra at different excitation wavelengths, and (c) time-resolved fluorescence.

Figure 5c shows the time-resolved fluorescence curve, resembling the IRF (instrument response function). Therefore, the precision of this measurement is low, but we estimate τ_fl_ to be ca. (0.7 ± 0.3) ns. Table 3 summarizes the time-resolved fluorescence results for this sample, including the radiative (*k*_r_) and nonradiative (*k*_nr_) rates.

**Table 3:** Fluorescence Quantum Yield, Fluorescence Lifetime, and Radiative and Nonradiative Rates for Molecule **A6**

	ϕ_fl_ (%)	τ_fl_ (ns)	*k*_r_ (×10^8^ s^–1^)	*k*_nr_ (×10^9^ s^–1^)
**A6**	14	0.7	2.2	1.4

The results for ⟨*r*⟩ and solvatochromism are presented in Figure 6. As can be seen, ⟨*r*⟩ in the first band remains approximately constant, indicating that the angle between the absorption and emission dipoles remains unchanged (Figure 6a). This suggests that this band corresponds to a single electronic transition. In contrast, ⟨*r*⟩ in the second band, around 280 nm, exhibits variable behavior, indicating the presence of multiple transitions in this region. These observations are consistent with the simulations in Figure 3c. It is important to note that ⟨*r*⟩ values remain within the expected range of 0.4 (for parallel or antiparallel absorption and emission dipole moments) to −0.2 (for perpendicular absorption and emission dipole moments). Additionally, when we added glycerol to the DMSO solution, increasing the viscosity, the ⟨*r*⟩ value increased and tended toward the *r*_0_ value in the absence of a rotational diffusion process. We can plot the Perrin equation by taking the average ⟨*r*⟩ values from the first band, as shown in Figure 6b. We believe that the linear behavior of this curve can be attributed to the very short τ_fl_ of **A6**, which allows us to approximate ⟨*r*⟩ by writing

6 $$\left\langle r\right\rangle =\frac{r_{0}}{1+\tau_{fl}/\Theta }\approx r_{0}\left(1-\frac{\tau_{fl}}{\Theta }\right)=r_{0}-\frac{r_{0}\tau_{fl}R}{V}\frac{T}{\eta }$$

where $\Theta =\frac{\eta V}{RT}$ is the rotational correlation time, *V* is the molar volume (which allows the determination of the Onsager radius), *R* is the gas constant, and *T* is the temperature.

**Figure 6:**
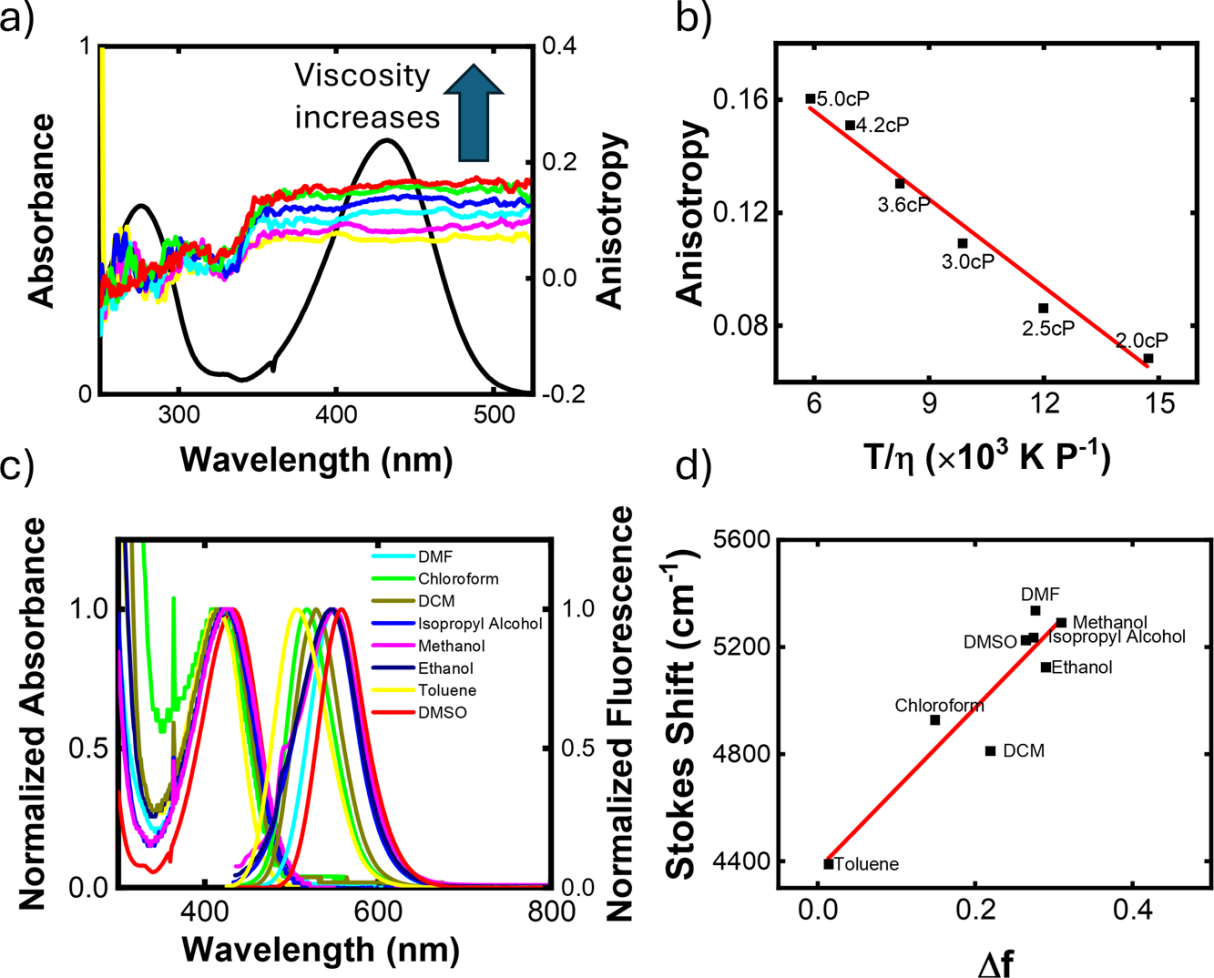
Anisotropy and solvatochromism results: (a) anisotropy as a function of excitation wavelength, (b) Perrin plot showing average anisotropy in the lower-energy band as a function of temperature and viscosity, (c) absorption and emission spectra in different solvents, and (d) Lippert–Mataga plot showing the Stokes shift versus orientational polarizability.

The linear coefficient of the graph in Figure 6b indicates that *r*_0_ = (0.218 ± 0.008), corresponding to an angle between the absorption and emission dipole moments of β = (33.4 ± 0.1)°. Since the system is excited from S_0_ to S_1_ and the photon is expected to be emitted during the transition from S_1_ to S_0_, the emission dipole moment should theoretically be equal to that of the absorption, resulting in *r*_0_ = 0.4. Thus, the value obtained from the linear adjustment of Figure 6b suggests that the molecule may present some structural mobility, adopting a different conformation in the excited state, which causes the dipole moments to differ. From the angular coefficient of the graph in Figure 6b, we obtained an Onsager radius of *a* = (7.8 ± 0.8) Å.

Figure 6c shows the absorption and emission spectra of molecule **A6** in different solvents. The variation in the emission peak is greater than that of the absorption one, as orientational polarizability varies, indicating that emission is more sensitive to the changes in the medium than the absorption. In addition, more polar solvents, such as DMSO, tend to shift the emission peak further toward the red region than less polar solvents, such as toluene. Figure 6d shows the Lippert–Mataga plot, and its angular coefficient gives a Δμ_01_ value of (12 ± 2) D. This represents a relatively large variation in dipole moment but is consistent with values found in the literature for similar molecules.^51^

### Transient Absorption

Figure 7 presents the TAS results for molecule **A4**. The positive signal in Figure 7a indicates excited-state absorption (ESA) for a highly energetic S_*n*_ state. Furthermore, we observed an intriguing dynamic behavior: a rapid increase in transient absorption (ΔAbs) within the first ∼20 ps, followed by a slower decay. This suggests that upon excitation of the molecule to a Franck–Condon (F) state—where the nuclear configuration remains unchanged—the system decays to a relaxed (R) state, likely involving a conformational change, as supported by the anisotropy measurements. Thus, immediately after the pump excitation, all excited species in the solution are assumed to occupy the F state. Subsequently, a relaxation process transfers population to the R state, which is believed to possess a higher ESA cross section. This evolution from F to R is represented by the rising exponential component, characterized by the negative (red line) curve in the decay-associated spectra (DAS) in Figure 7b. In contrast, the decay exponential component associated with the R state is depicted by the positive (black line) curve in the DAS. Then, the less stable F state quickly decays with a lifetime of 3.9 ps to a more stable R state, which has a lifetime of 1.6 ns. The transition in the ESA spectrum from F to R is likely responsible for the slight red shift observed in Figure 7d. Moreover, the modulation of the decay signal in the time domain (Figure 7c) by a complex oscillatory pattern can be attributed to the vibrational coherence of the compound excited by the pump.^52^

**Figure 7:**
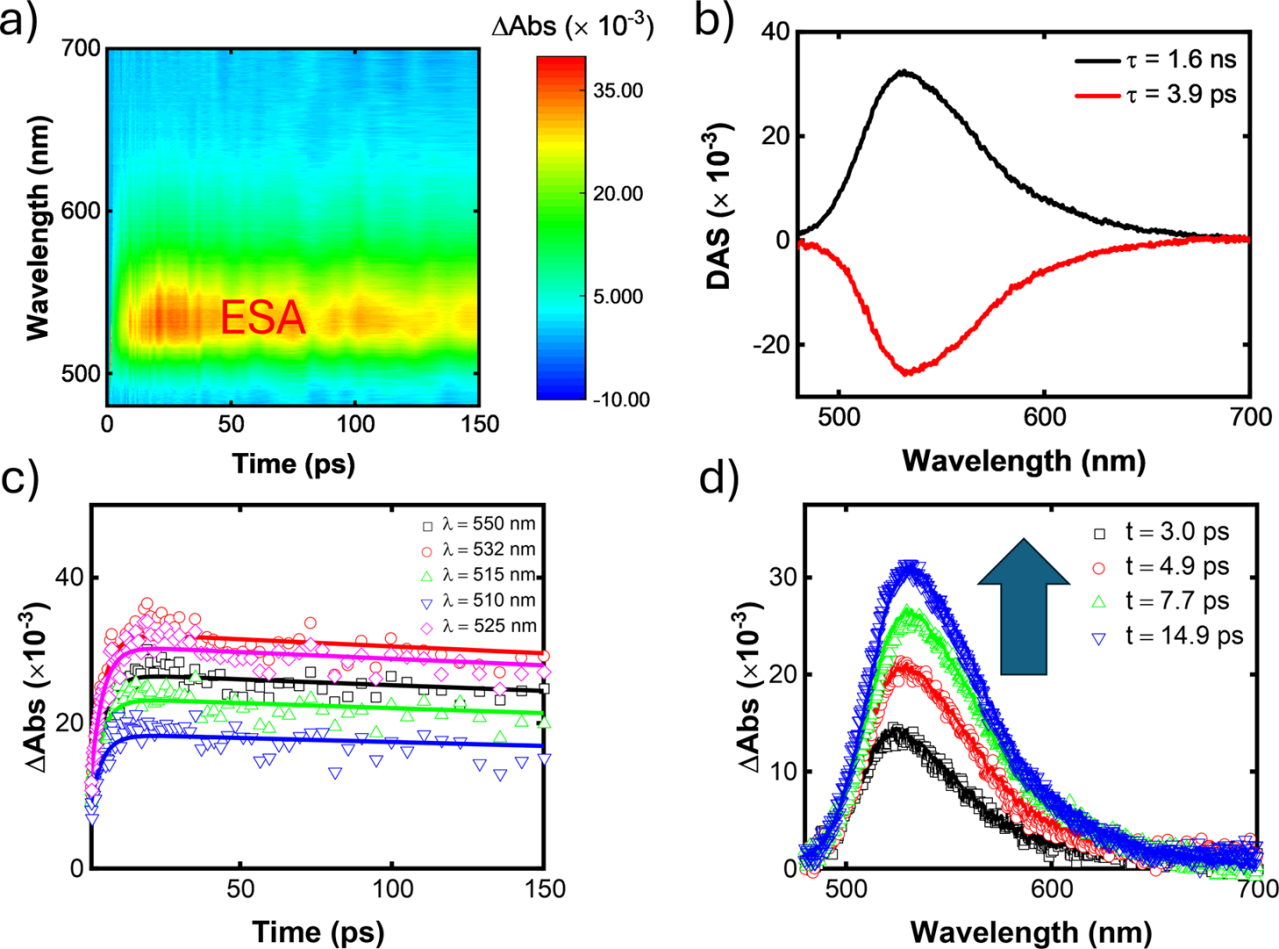
Transient absorption for **A4**: (a) two-dimensional colormap, (b) decay-associated spectra, (c) transient absorption as a function of time delay for selected wavelengths, and (d) transient absorption spectra for some time delay.

The region where ESA was observed overlaps with the range exhibiting the highest D-2PACS values, which may indicate a possible contribution of the ESA to the measured OA Z-scan signal. To assess this possibility, we analyzed the 2PA coefficient (β) as a function of peak intensity.^53–55^ A constant β is indicative of pure 2PA, whereas a variation with intensity suggests the involvement of additional processes, such as 2PA-assisted ESA. We observed that β remains approximately constant in the studied spectral region, within experimental error, as shown in Figure 8. These results suggest that any contribution from ESA to the OA Z-scan signal is likely minor, particularly given the use of femtosecond laser pulses, which reduces the probability of such effect.

**Figure 8:**
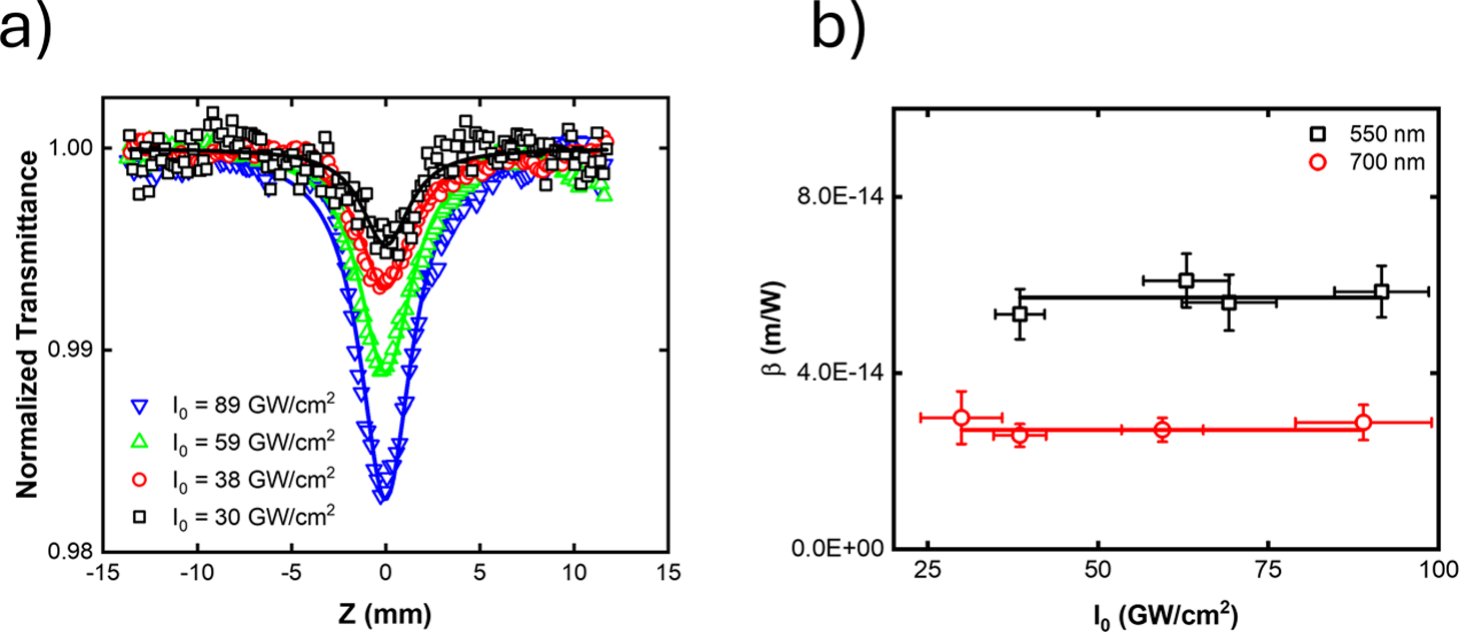
(a) OA Z-scan measurements at 700 nm and (b) 2PA coefficients of molecule **A4** at different peak intensities.

All other samples exhibited TAS similar to that of **A4**, except **A6**, which, in addition to ESA, presents stimulated emission (SE), which contributes to a negative ΔAbs value, as we can see in Figure 9. The global analysis shows an excited-state lifetime of approximately 0.63 ns, consistent with the previously obtained τ_fl_. Figure 9d shows that the minimum undergoes a red shift over time, reaching 558 nm, which corresponds to the fluorescence emission peak. This shift can be associated with the differences in the emissions between the F and R species and/or the solvation effect in response to the excited-state permanent electric dipole moment of this sample. In Figure 9c, we see an ESA in which ΔAbs initially rises quickly and then decays slowly, as previously discussed for molecule **A4**. The stimulated emission decays rapidly at 543 and 558 nm, indicated by the progressively less negative ΔAbs values, followed by a slower decay. This behavior could result from the superposition of the ESA and SE or SE originating from both F and R states. Indeed, in Figure 9c, at a more distant wavelength (613 nm) where only the stimulated emission of the R state is expected, we observe that SE initially increases rapidly, indicated by increasingly negative ΔAbs values, representing the population of R state and then it decays slowly toward zero, representing the depletion of this state.

**Figure 9:**
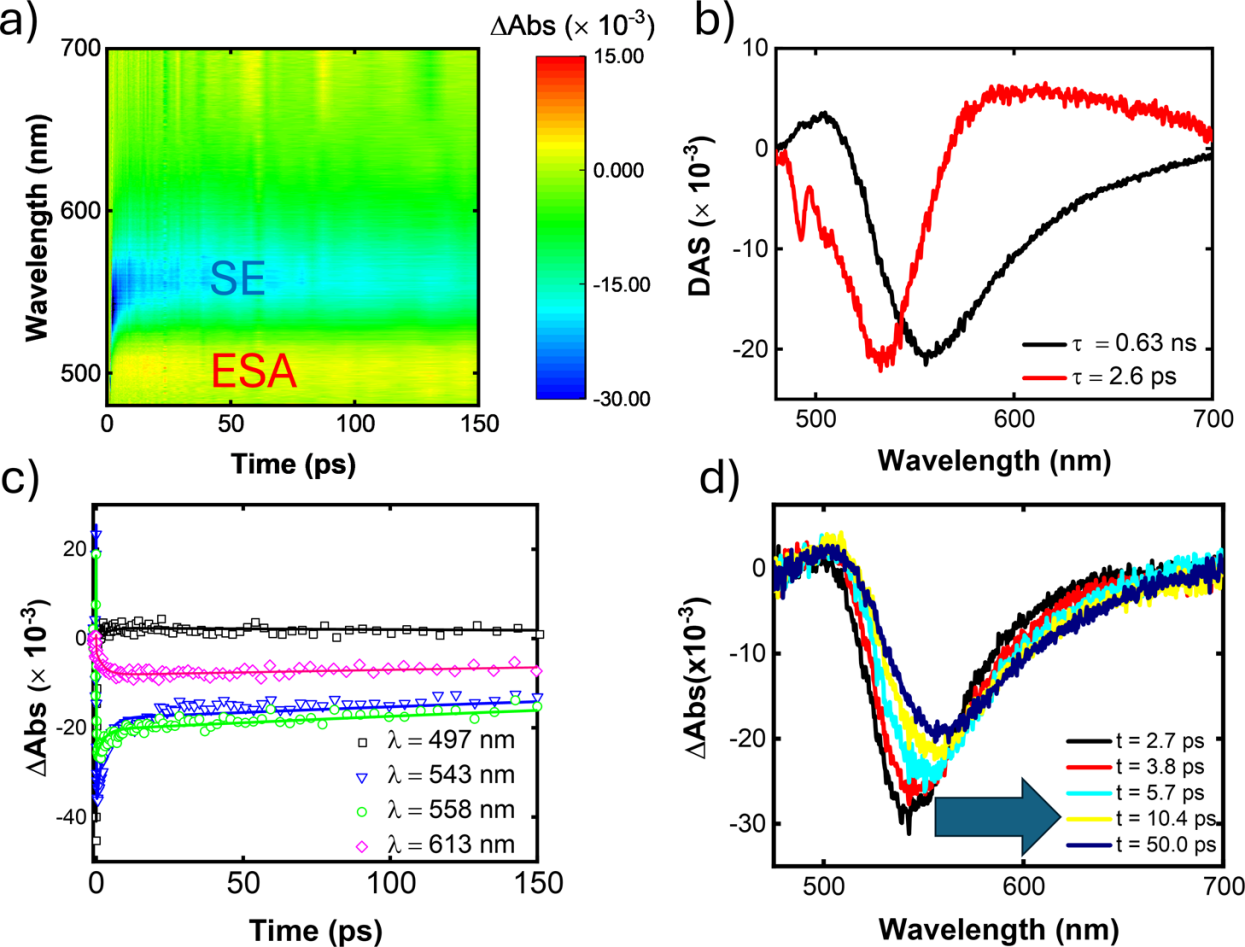
Transient absorption for **A6**: (a) 2D colormap, (b) decay-associated spectra, (c) transient absorption as a function of time delay for selected wavelengths, and (d) transient absorption spectra for some time delay.

To support this hypothesis of conformational relaxation, we performed geometry optimization for the first excited state. The samples yielded similar results, as illustrated in Figure 10 for molecule **A6**. The analysis reveals that these molecules undergo torsion, as schematized in Figure 10a, with pronounced charge separation (Figure 10a), classifying this first excited state as a twisted intramolecular charge transfer (TICT) state. These states are known for having a high permanent electric dipole moment, which justifies the significant Stokes shift in the fluorescence spectra. Furthermore, charge transfer (CT) results in a low orbital overlap (Figure 10a), which leads to a reduced transition dipole moment. This phenomenon helps explain the low radiative decay rate, which, when combined with an elevated nonradiative decay rate, results in the molecules being “non-fluorescent”, i.e., having a low ϕ_fl_.^56,57^

**Figure 10:**
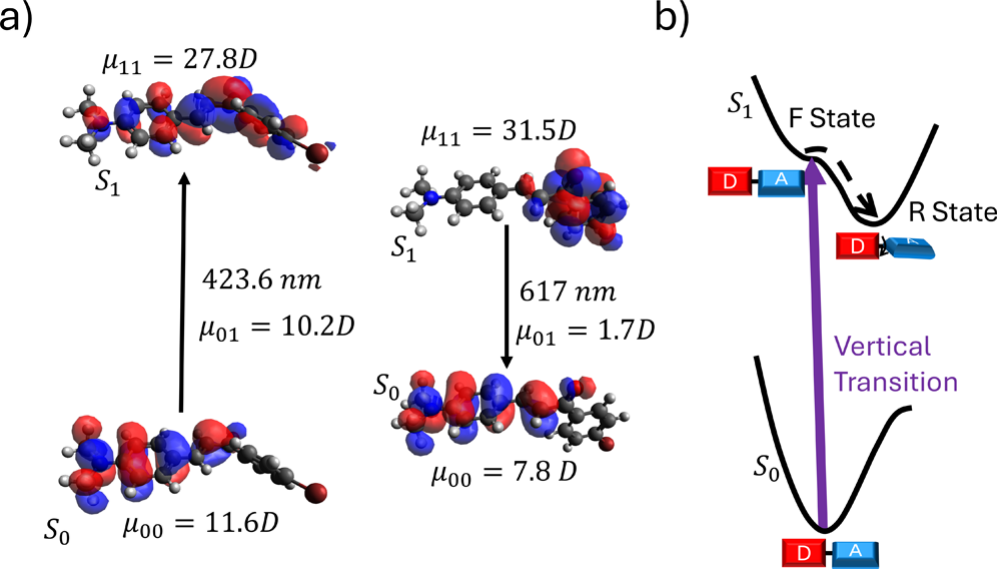
Geometry optimization of the first excited state for molecule **A6**: (a) orbitals and electric dipole moments from the ground state (S_0_) to the excited state (S_1_), including the transition wavelength and (b) schematic diagram representing the vertical transition and the relaxation process of this molecule.

## Conclusions

In this work, we measured D-2PACS using the OA Z-scan technique on various bromochalcone derivatives. In general, the samples exhibited low 2PACS, mainly in the lower-energy band, with the higher-energy states being more easily accessed by two photons. However, some molecules showed strong nonlinear absorption in the lower-energy band, indicating promising potential for applications in NLO devices. Molecule **A6**, for example, exhibited a D-2PACS of approximately 90 GM and was the only one with a significant fluorescent quantum yield with a large Stokes shift, making it attractive for nonlinear fluorescence microscopy. The enhancement in nonlinear absorption observed in some samples can be attributed to the resonant donor character of the substituent groups, which are also responsible for the largest bathochromic shifts. Furthermore, TAS measurements revealed ESA in the spectral region corresponding to the highest D-2PACS, which may indicate a small contribution of 2PA-assisted ESA in the OA Z-scan signal, although significant changes of β with peak intensity were not observed. The TAS data also exhibited an interesting dynamic characterized by a rising exponential component, indicating the population of another excited state associated with the formation of a TICT state.

## Supplementary Material



## Notes

Procedures for measuring linear photophysical properties, including one-photon absorption, fluorescence emission, fluorescence quantum yield, solvatochromism, and anisotropy; open-aperture Z-scan technique to measure the degenerate two-photon absorption cross section; femtosecond transient absorption technique; quantum chemical calculation procedures; experimental and computational results of all samples, such as the transient absorption analysis and the theoretical one-photon absorption spectra; and references (PDF)
