# Supplementary material for: Two-Photon Absorption and Dynamics of Excited States in Bromochalcone Derivatives
Source: J Phys Chem A. 2025 Sep 30;129(40):9119–28. doi: 10.1021/acs.jpca.5c02748 (PMC12516734; doi:10.1021/acs.jpca.5c02748)
Supplement: Supplementary file 1 [file jp5c02748_si_001.pdf]

# Supporting Information

## Two-Photon Absorption and Dynamics of Excited States in Bromo-Chalcone Derivatives

Nathan B. Marucci[1], João V. P. Valverde[1], Gabriel de O. Campos[1], Eli S. A. Ducas[2,3], Pablo J. Gonçalves[2,4,5], Leonardo De Boni[1], Cleber R. Mendonça[1]

[1]Photonics Group, São Carlos Institute of Physics, University of São Paulo, CP 369, 13566-590 São Carlos, SP, Brazil.

[2]Institute of Chemistry, Federal University of Goiás, 74690-900, Goiânia, GO, Brazil

[3]Water Treatment and Sanitation Company of Goiás, Superintendence of Construction Management, 74805-100, Goiânia, GO, Brazil

[4]Institute of Physics, Federal University of Goiás, 74690-900, Goiânia, GO, Brazil.

[5]Center of Excellence in Hydrogen and Sustainable Energy Technologies (CEHTES), 74.690-631, Goiânia, GO, Brazil.

# SI1. Linear Photophysical properties

To investigate the linear photophysical properties of the compounds, we prepared solutions in dimethyl sulfoxide (DMSO) at concentrations of approximately $10^{-4}$M.

## SI1.1 Absorption and Emission Spectra

One-photon absorption (1PA) spectra were recorded using a UV-Vis spectrophotometer (UV-1800, Shimadzu) with a 2 mm path length quartz cuvette, while fluorescence spectra were obtained with a spectrofluorometer (F-7000, Hitachi), using a 1 cm path length cuvette.

## SI1.2 Transition dipole moment

To estimate the transition dipole moments from the ground state to the excited state ($\mu_{0f}$), we decompose the UV-Vis linear absorption spectra into Gaussians and apply the following equation:

$$\left\langle \sigma_{0f}^{(1)} \right\rangle = \frac{\pi\omega}{3n\epsilon_0 c} \frac{|\boldsymbol{\mu}_{0f}|^2}{\hbar} \rho_f(\omega_{0f} = \omega) \tag{1}$$

where $\sigma_{0f}^{(1)}$ is the 1PA cross-section from the ground state $|0\rangle$ to the final state $|f\rangle$, $n$ is the refractive index, $\epsilon_0$ is the vacuum permittivity, $c$ is the speed of light, $\hbar$ is the reduced Planck constant, and $\rho_f$ is the line shape of the final state.

## SI1.3 Fluorescence Quantum yield

We determine the fluorescence quantum yield ($\phi_{fl}$) using the well-known comparative method (1,2), through Eq. ( 2 ). We used the molecule of chalcone reported in (3) with $\phi_f = 71\%$ in DMSO as reference and measured the emission and absorption spectra under the same experimental conditions.

$$\phi_{fl} = \phi_{flR} \times \frac{Int}{Int_R} \times \frac{1-10^{-Abs_R}}{1-10^{-Abs}} \times \frac{n^2}{n_R^2} \quad (2)$$

In the equation above, the subscript $R$ refers to the reference sample, $Int$ is the area under the fluorescence curva, $Abs$ is the absorbance and $n$ is the refractive index.

## SI1.4 Fluorescence Lifetime

For fluorescence lifetime ($\tau_{fl}$) measurements, we used the third harmonic ( $343\ nm$) of the Pharos laser system (Light Conversion, PH1) with a pulse duration of approximately $220\ fs$ and a repetition rate of $300\ Hz$. A converging lens focused the beam, with the sample in a $2\ mm$ cuvette placed slightly beyond the focal point. The fluorescence intensity was collected perpendicularly to the excitation through an optical fiber and guided to a photodetector. To determine the instrument response function (IRF), we used a scattering glass.

## SI1.5 Steady-state anisotropy

The steady-state anisotropy, $\langle r\rangle$ , was measured using a fluorimeter (F-7000 model, Hitachi) equipped with two polarizers: one controlling the excitation polarization and the other the emission polarization. Emission intensity was recorded as a function of excitation wavelength for four polarization configurations: VV, VH, HV. In these notations, the first letter represents the excitation polarization (vertical or horizontal), while the second indicates the emission polarization. We determine the $\langle r\rangle$ using (4,5):

$$\langle r\rangle = \frac{I_{VV} - GI_{VH}}{I_{VV} + 2GI_{VH}} \quad (3)$$

with $G = \frac{I_{HV}}{IHH}$ being an experimental apparatus correction factor.

Anisotropy measurements were repeated after incrementally adding glycerol. The results were used to construct a Perrin plot (Eq. ( 4 )) and determine the Onsager radius, $a$, supposing the fluorophore with a spheric symmetry.

$$\langle r \rangle = \frac{r_0}{1+\tau_{fl}/\Theta} \quad \text{with} \quad \Theta = \frac{\eta V}{RT} \tag{4}$$

where $r_0$ is anisotropy in absence of rotational diffusion processes, $\eta$ is viscosity, $V$ is the molar volume (which is supposed to be of a sphere), $R$ is the ideal gas constant, and $T$ is temperature.

The viscosity of the DMSO-glycerol mixture was determined using the Arrhenius equation (6):

$$\ln \eta = x_1 \ln \eta_1 + x_2 \ln \eta_2 \tag{5}$$

where $x_i$ is the molar fraction of the *i-th* component of the mixture.

## SI1.6 Solvatochromism and Permanent Dipole Moment Difference

To measure solvatochromism, we dissolved the sample in solvents with different polarities and measured the linear absorption and emission spectra to plot the Lippert-Mataga equation (Eq. ( 6 )) and determine the $\Delta\boldsymbol{\mu}_{01}$ value.

$$\bar{\nu}_A - \bar{\nu}_F = \frac{2\,|\Delta\boldsymbol{\mu}_{01}|^2}{a^3 hc}\left[\frac{\epsilon - 1}{2\epsilon + 1} - \frac{n^2 - 1}{2n^2 + 1}\right] + cte \tag{6}$$

# SI2. Open-Aperture Z-scan Technique

For the nonlinear optical measurements, we employed $10^{-2}$M solutions in DMSO. Figure S2Figure S1 shows a schematic of the Z-scan technique. The light source is a Yb:KGW laser system (PHAROS PH1, Light Conversion) centered at $1030\ nm$, with a repetition rate of $750\ Hz$ and a pulse duration of approximately $220\ fs$. This system pumps an optical parametric amplifier (Orpheus, Light Conversion), which allows us to tune the excitation wavelength from the UV to near-infrared. A spatial filter was used to ensure an approximately Gaussian beam profile, as supposed in the theory (7). This filter consisted of a small slit to generate a diffraction pattern, and a pinhole was used to select the central mode. A portion of the laser beam was sent to a reference detector by a beamsplitter to correct any laser fluctuation. The remaining portion was focused by a convergent lens, and the sample was translated along the beam propagation axis near the focal region. The transmittance signal was measured with a second detector and filtered by a lock-in amplifier.

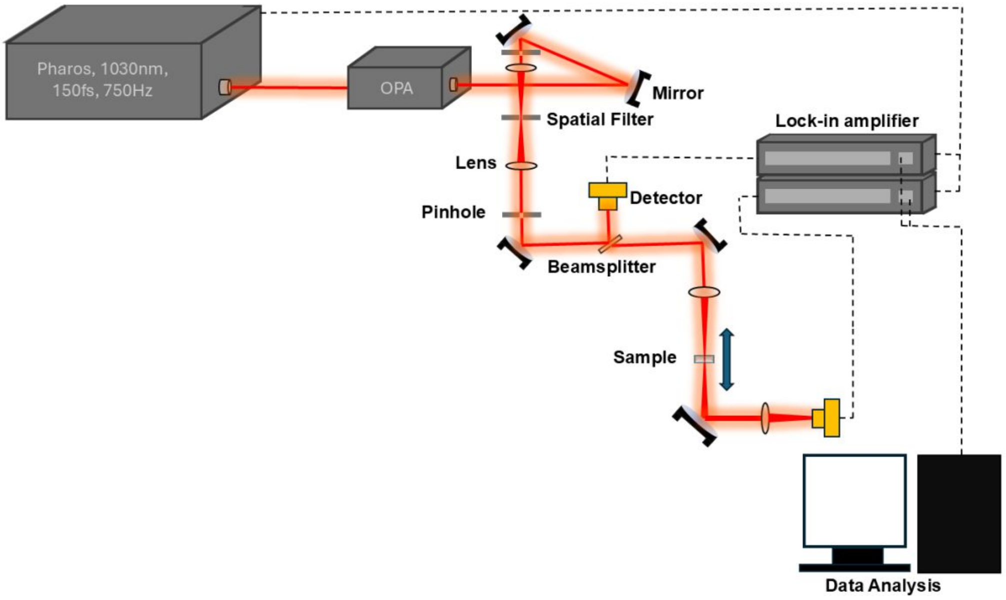


FIGURE S1: SCHEME OF Z-SCAN SETUP

To fit the normalized transmittance, we used the following equation:

$$T(z) = \sum_{m} \frac{\left[-q_0(z,0)\right]^m}{(m+1)^{3/2}} \quad (7)$$

With

$$q_0(z,0) = \frac{\beta I_0 L}{1 + \left(\frac{z}{z_r}\right)^2} \quad (8)$$

where $\beta$ is the two-photon absorption coefficient, $I_0$ is the peak intensity, $L$ is the sample thickness, and $z_r$ is the Rayleigh length. For small, normalized transmittance changes, we truncated Eq. ( 7 ) at $m = 3$ to fit the experimental data. Typically, the change in normalized transmittance is $\Delta T \sim 1\%$, as shows Figure S2, which justifies this approximation.

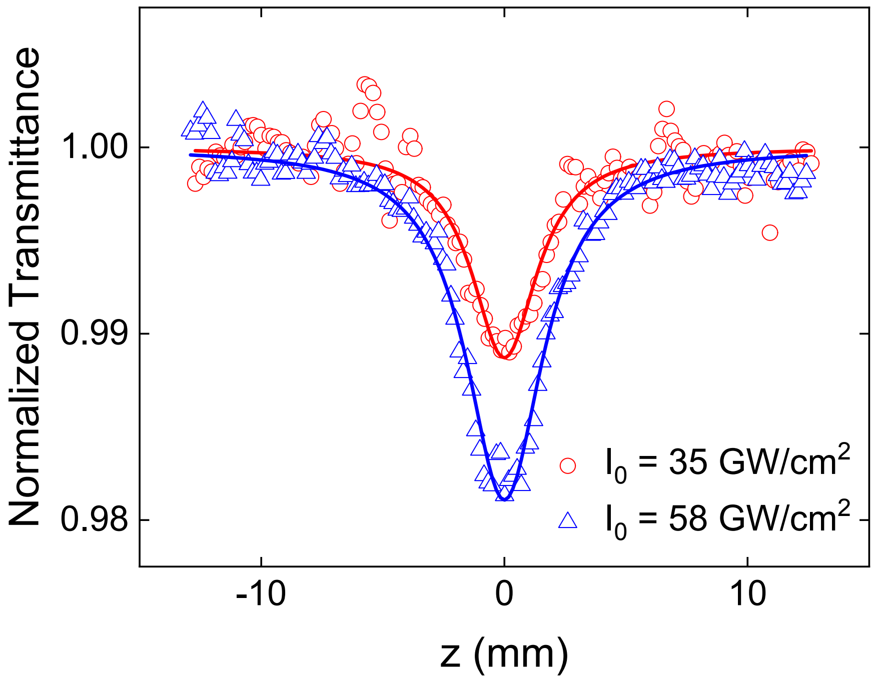


FIGURE S2 - Z-SCAN SIGNATURE FOR MOLECULE A5 AT 700 NM

We determined the degenerate two-photon absorption cross-section (D-2PACS) using:

$$\sigma^{(2)}(\lambda) = \frac{\hbar\omega\, \beta(\lambda)}{N} \quad (9)$$

where $\hbar\omega$ is the photon energy and $N$ is the density of molecules in solution.

# SI3. Transient Absorption Technique

The laser system used in the transient absorption technique (8) was the same as described above. The pump beam is the output of the OPA. On the other hand, the probe

beam is a white light supercontinuum generated by focusing the laser beam onto a sapphire crystal. Transient absorption was measured by comparing the spectrum of the probe beam before and after the incidence of the pump. To achieve this condition, we used a chopper as demonstrated in Figure S3. The measurements were performed with the pump and probe polarization set at the magic angle. For the sample A6, we set the pump beam wavelength to 430 $nm$, while for the others we set it to 340 $nm$. For global analysis, we used Glotaran software. (9)

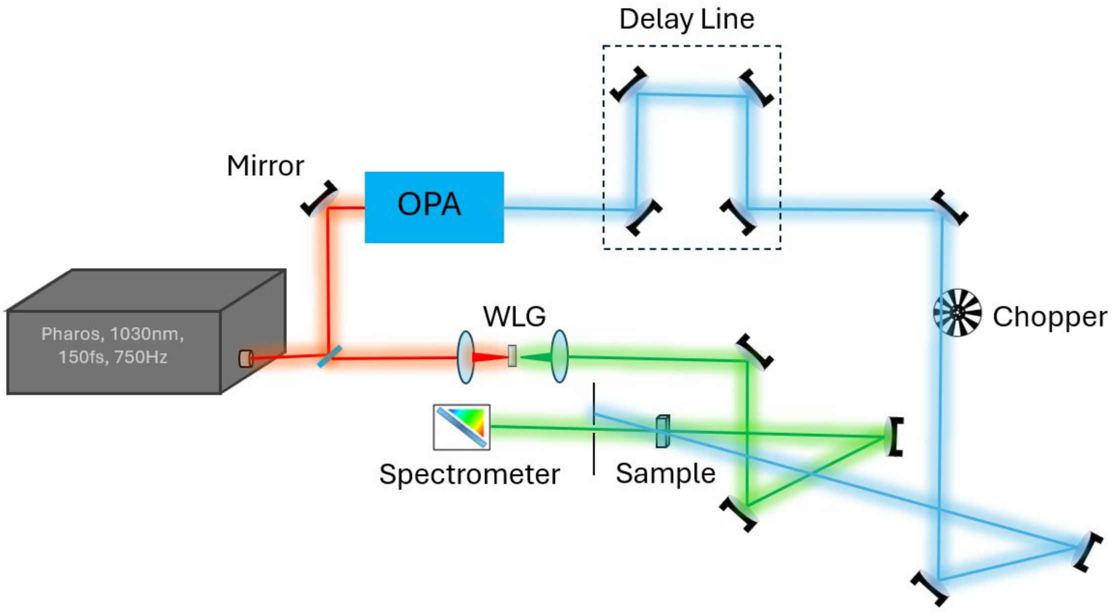


FIGURE S3: SCHEME OF PUMP-PROBE APPARATUS

# SI4. Quantum Chemical Calculations

Quantum chemical calculations were performed using the Gaussian 09 software to investigate the electronic and structural properties of the compounds. Geometry optimization was carried out at the DFT level, and based on the optimized structures, electronic transition characteristics were computed using the TD-DFT approach. The calculations employed the PBE1PBE functional and the 6-311++G(d,p) basis set. To account for solvent effects in DMSO, the Polarizable Continuum Model (PCM) with the Integral Equation Formalism (IEF-PCM) was applied.

Natural transition orbitals (NTOs) for the first electronic transitions were analyzed using Multiwfn software. NTOs were computed for all samples except A4, A5 and A6,

which exhibited high contribution coefficients. The simulated spectra were generated using a full width at half maximum (FWHM) of $0.33\ eV$ for all transitions.

# SI5. Experimental results

## SI5.1 Decomposition of the linear spectra into Gaussian Functions

Figure S4 exhibits the results of the decomposition of the linear spectral of the samples into Gaussian functions. In these decompositions, we used up to three functions to adjust the experimental data.

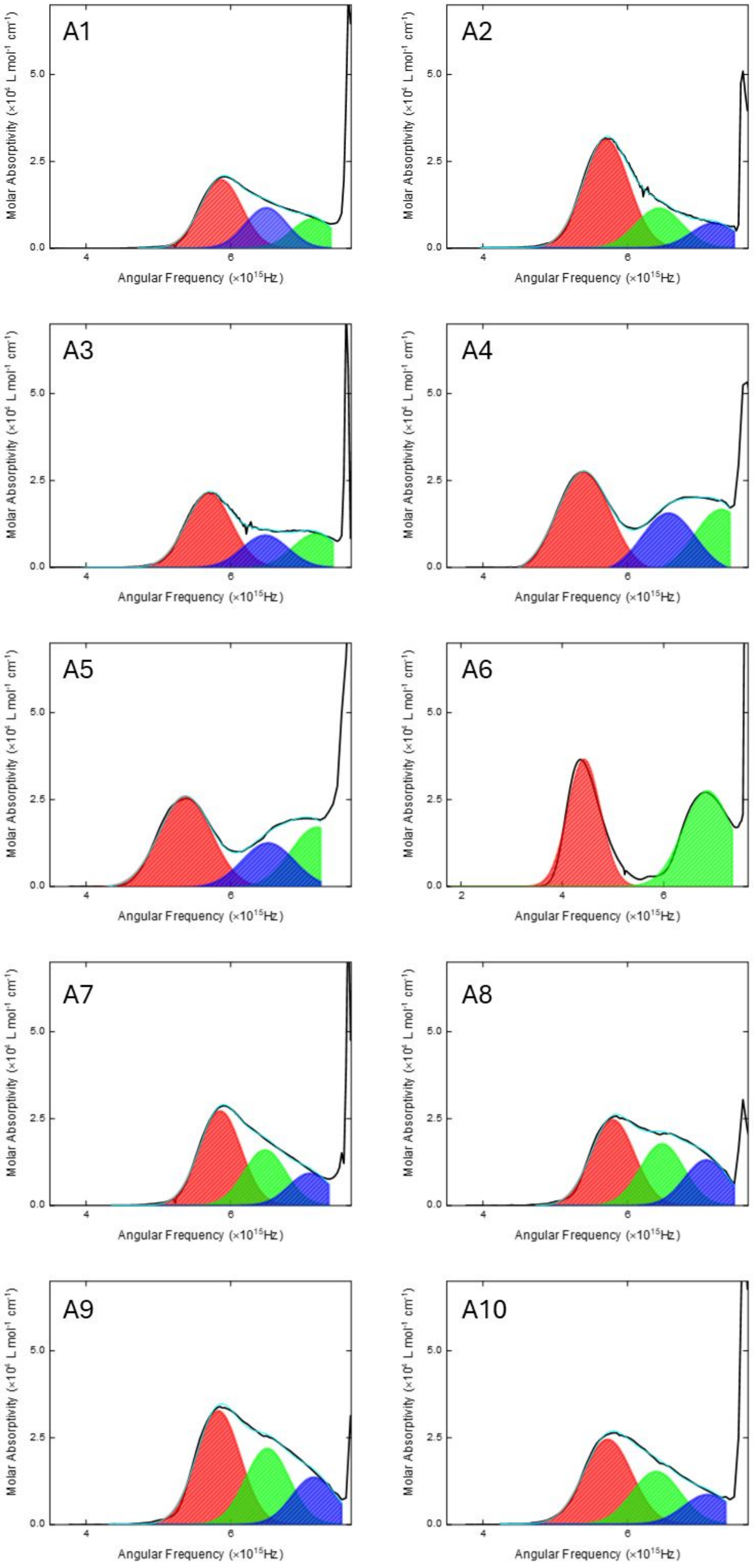


FIGURE S4: DECOMPOSITION OF LINEAR ABSORPTION SPECTRA INTO GAUSSIAN FUNCTIONS

## SI5.2 Transient absorption

Figure S5 presents an example of transient absorption result for molecule A2. This molecule exhibits the same behavior as molecule A4, discussed in the paper, but with a shorter lifetime.

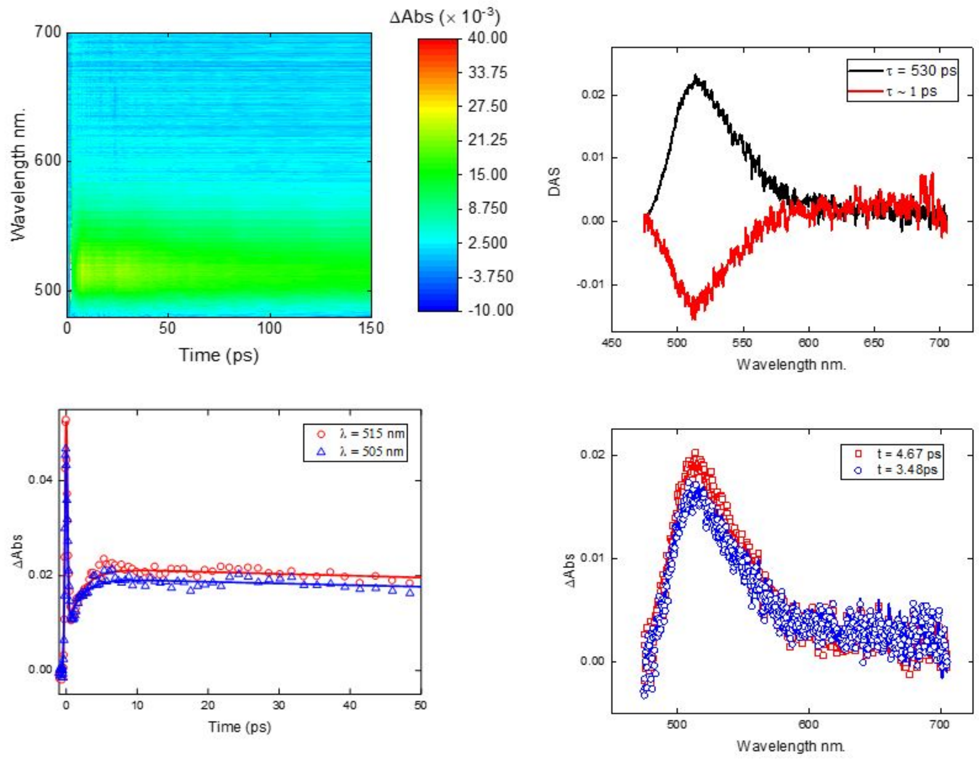


FIGURE S5: TRANSIENT ABSORPTION RESULTS FOR MOLECULE A4

Table S1 presents the lifetimes of the F and R states obtained though global analysis. The halogen-substituted samples also exhibit a rising exponential component but with a very short lifetime, which makes the fits less accurate. On the other hand, the A1 and A10 samples likely have such short lifetimes that the rising exponential cannot be observed.

TABLE S1: LIFETIMES OF THE EXCITED STATES

| | $A1$ | $A2$ | $A3$ | $A4$ | $A5$ | $A6$ | $A7$ | $A8$ | $A9$ | $A10$ |
|---|---|---|---|---|---|---|---|---|---|---|
| $\tau_F$ (ps) | - | 1.0 | 1.0 | 3.9 | 4.4 | 2.6 | < 1 | < 1 | < 1 | - |
| $\tau_R$ $(ps)$ | 310 | 530 | 530 | 1588 | 1409 | 630 | 301 | 414 | 432 | 614 |

To understand the decay associated spectra of these samples, we assume that the F and R states can absorb a photon to reach a more energetic state, therefore we can write the transient absorption as

$$\Delta Abs(\lambda,t) \propto \sigma_{ESA}^{F}(\lambda) n_F(t) + \sigma_{ESA}^{R}(\lambda) n_R(t) \tag{10}$$

Considering the schematic of Figure S6, we can write the rate equations as:

$$\dot{n}_F = -(k_1 + k_2) n_F \tag{11}$$

$$\dot{n}_R = (k_1 + k_2) n_F - k_3 n_R \tag{12}$$

whose solutions are of the form:

$$n_F(t) = n_F(0) e^{-(k_1+k_2)t} \tag{13}$$

$$n_R(t) = -\frac{k_3}{k_1 + k_2 - k_3} n_F(0) e^{-(k_1+k_2)t} + \frac{k_3}{k_1 + k_2 - k_3} n_F(0) e^{-k_3 t} \tag{14}$$

By substituting Eq. ( 13 ) and Eq. ( 14 ) into Eq. ( 10 ), we obtain:

$$\Delta Abs(\lambda,t) \propto \left[\sigma_{ESA}^{R}(\lambda) \frac{k_3}{k_1 + k_2 - k_3} n_F(0)\right] e^{-k_3 t} + \left[-\sigma_{ESA}^{R} \frac{k_3}{k_1 + k_2 - k_3} n_F(0) + \sigma_{ESA}^{F}(\lambda) n_F(0)\right] e^{-(k_1+k_2)t} \tag{15}$$

Thus, except for the ESA attributed to F state, the decay associated spectrum (DAS) of $k_3$ ($530\ ps$) is opposite of $(k_1 + k_2)$ ($\sim 1 ps$), which agrees with Figure S5.

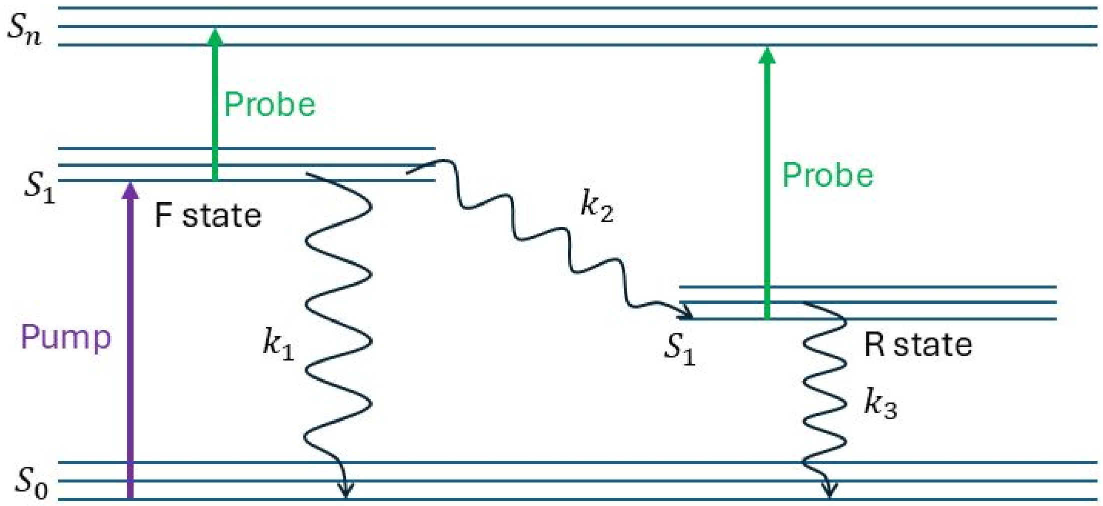


FIGURE S6: SCHEMATIC OF THE DYNAMICS OF EXCITED STATES

## SI5.3 Geometry Optimization

Figure S7 shows the results of geometry optimization of the ground state for each sample.

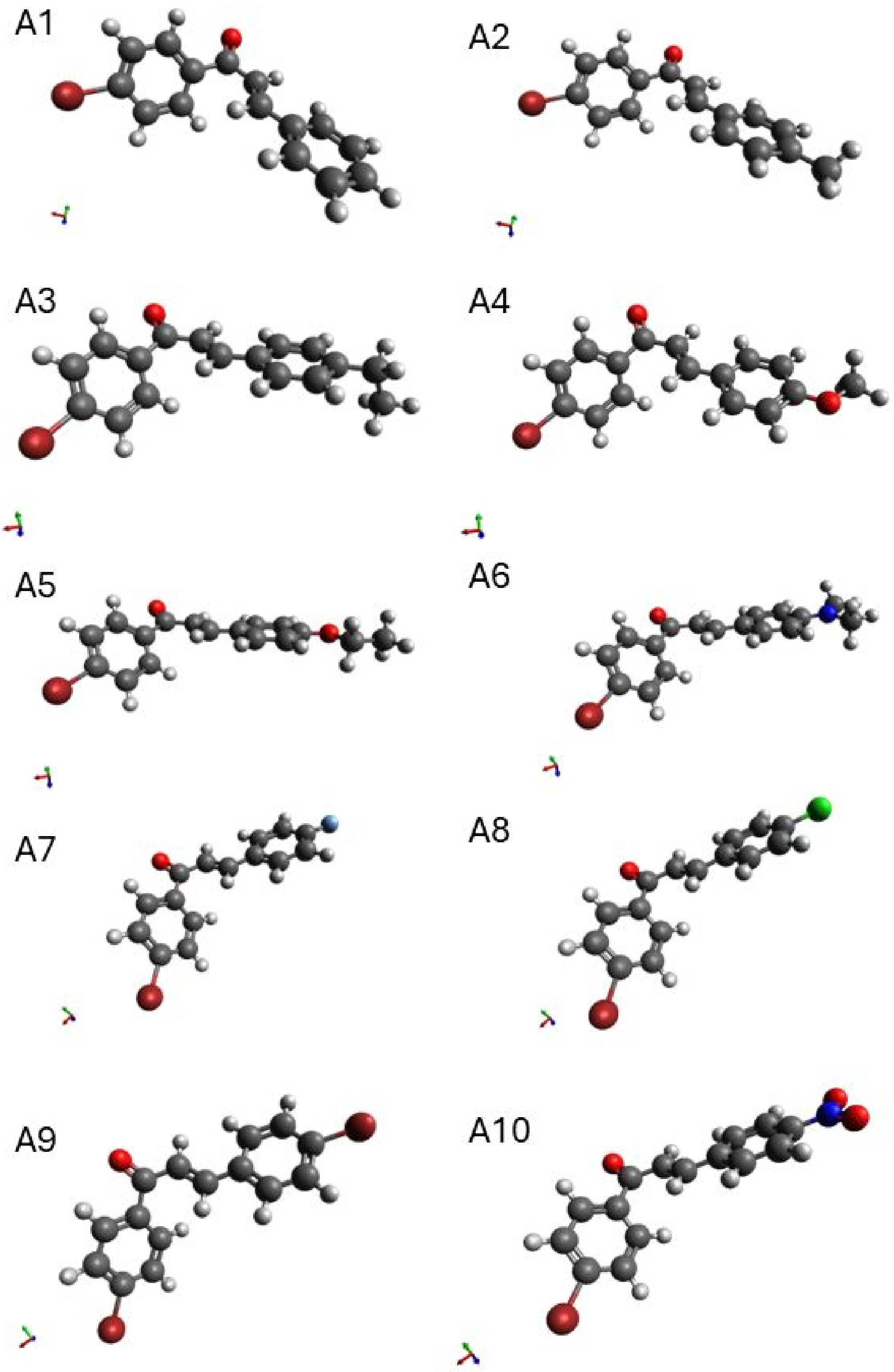


FIGURE S7: GEOMETRY OPTIMIZATION RESULTS FOR ALL MOLECULES

## SI5.4 Time-dependent simulations

The orbitals involved in the first excited states are shown in Figure S8, Figure S9 and Figure S10.

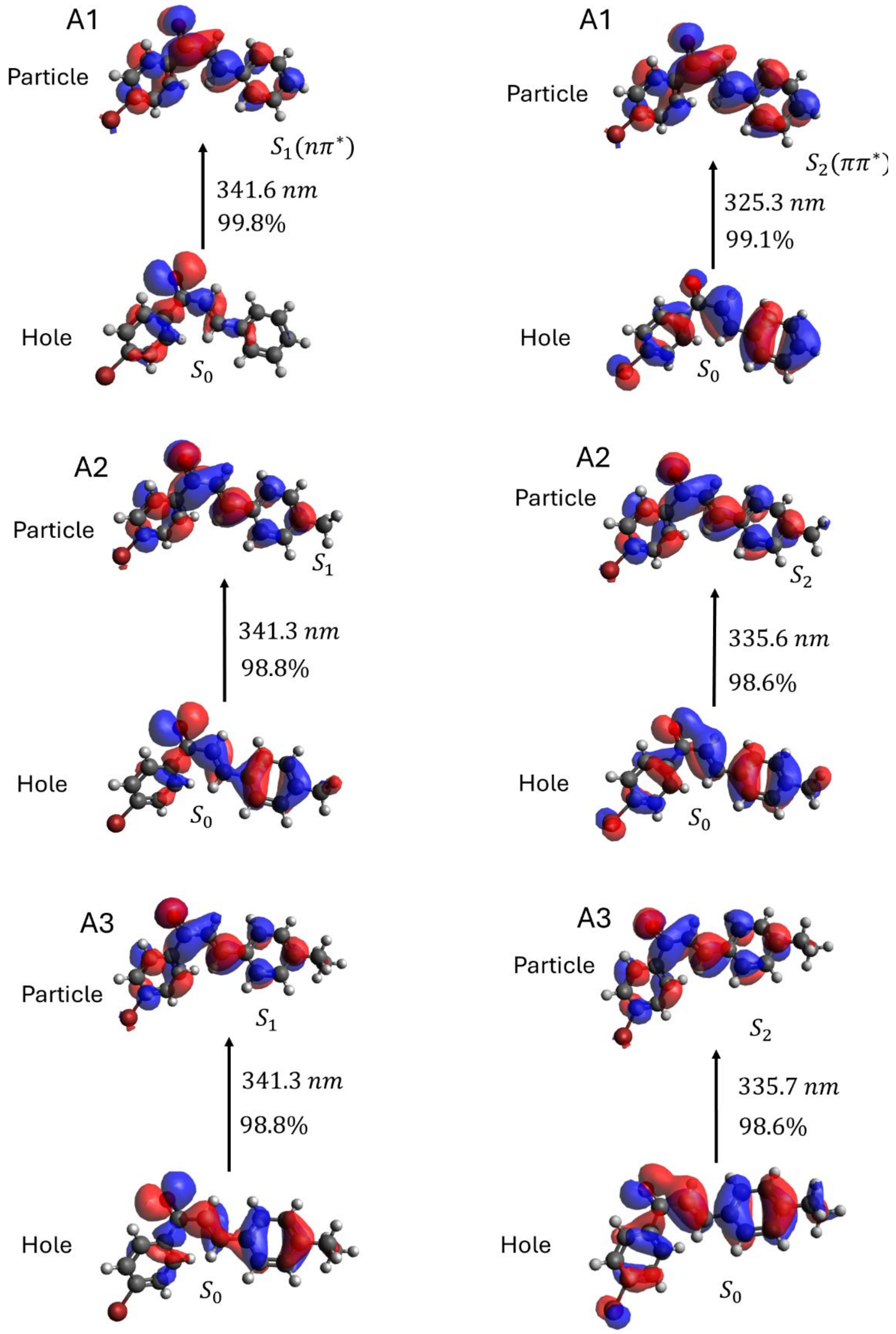


FIGURE S8: ORBITALS INVOLVED ON THE FIRST EXCITED STATES TRANSITIONS FOR A1, A2 AND A3 MOLECULES

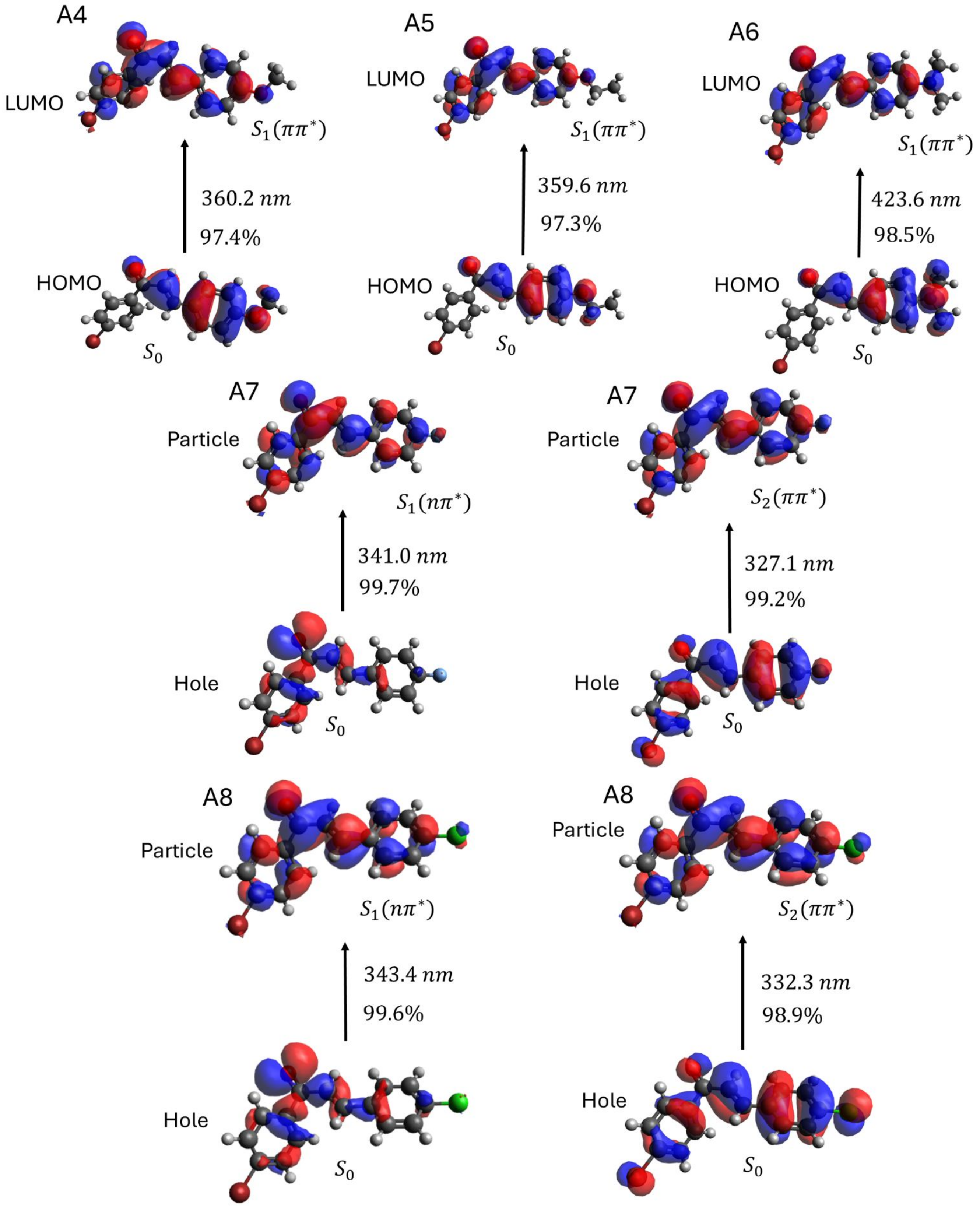


FIGURE S9: ORBITALS INVOLVED ON THE FIRST EXCITED STATES TRANSITIONS FOR A4, A5, A6, A7 AND A8 MOLECULES

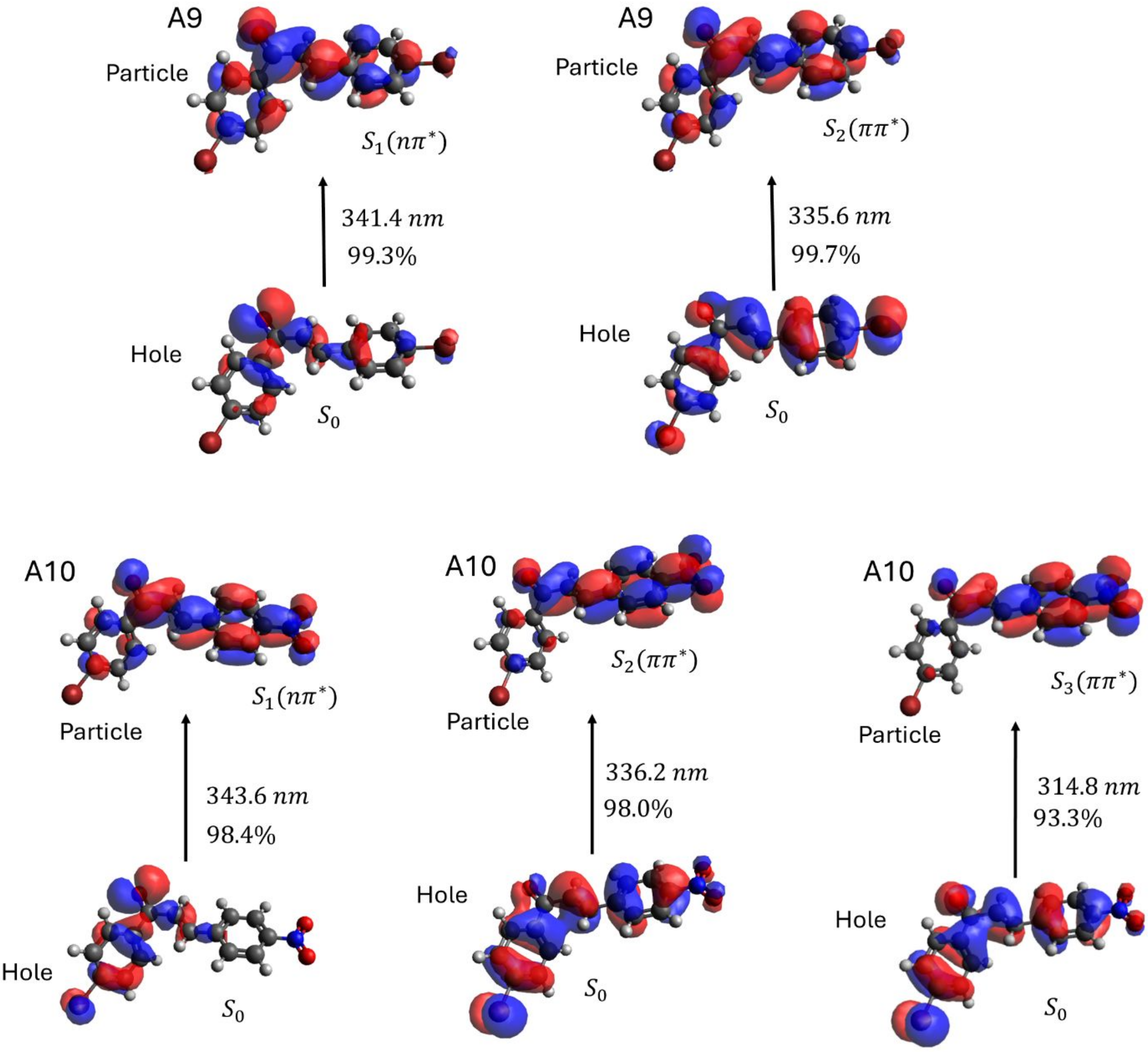


FIGURE S10: ORBITALS INVOLVED ON THE FIRST EXCITED STATES TRANSITIONS FOR A9 AND A10 MOLECULES

Figure S11 presents the theoretical one-photon absorption spectra for all samples.

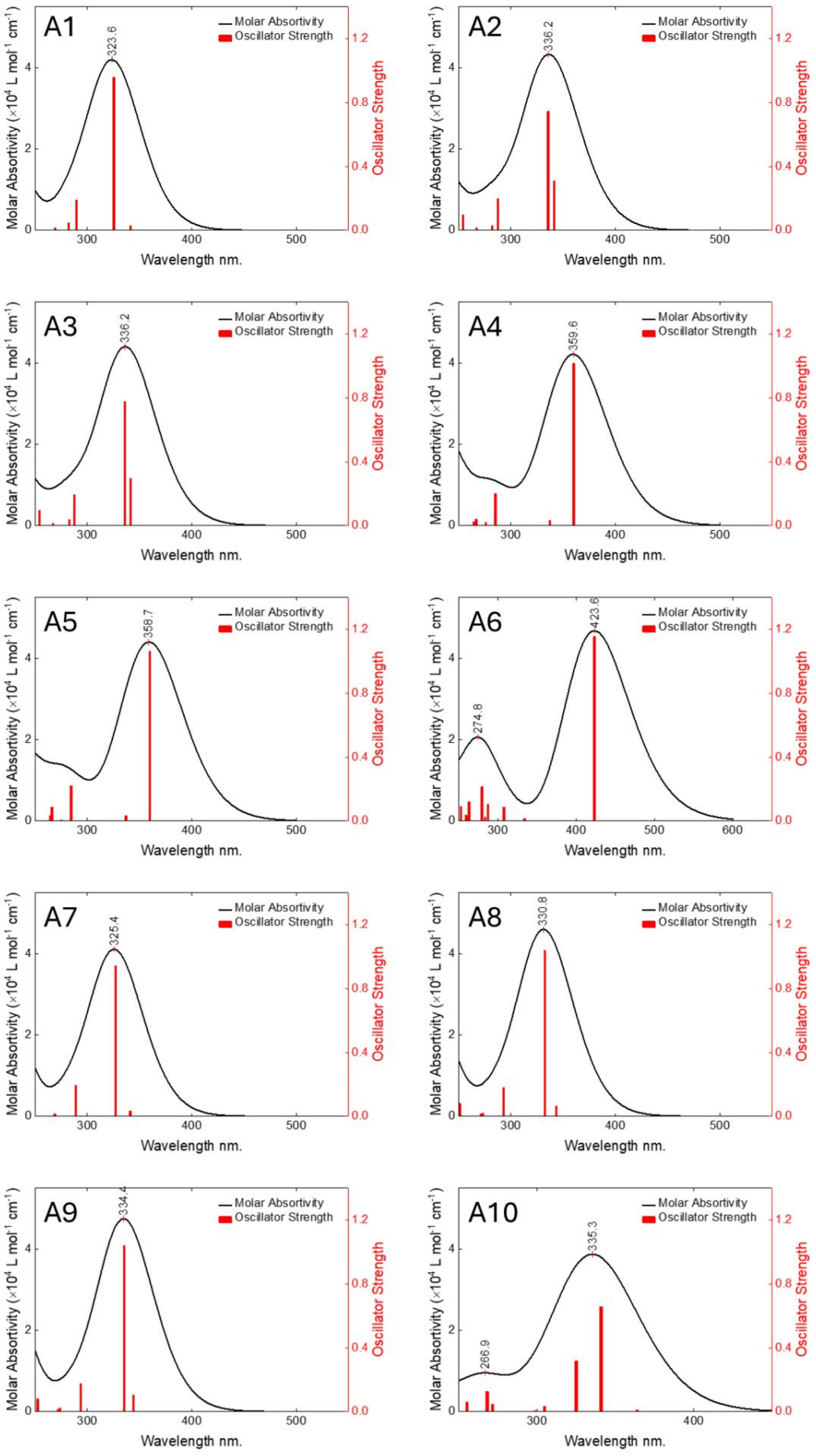


FIGURE S11: STIMULATED ONE-PHOTON ABSORPTION SPECTRA

# SI6. References


[1] CROSBY, G. A.; DEMAS, J. N. Measurement of photoluminescence quantum yields. Review. **The Journal of Physical Chemistry**, v. 75, n. 8, p. 991–1024, 1 abr. 1971.

[2] WILLIAMS, A. T. R.; WINFIELD, S. A.; MILLER, J. N. Relative fluorescence quantum yields using a computer-controlled luminescence spectrometer. **The Analyst**, v. 108, n. 1290, p. 1067, 1983.

[3] PELOSI, A. G. et al. Two-Photon Absorption and Multiphoton Excited Fluorescence of Acetamide-Chalcone Derivatives: The Role of Dimethylamine Group on the Nonlinear Optical and Photophysical Properties. **Molecules**, v. 28, n. 4, p. 1572, 6 fev. 2023.

[4] LAKOWICZ, J. R. Fluorescence Anisotropy. Em: **Principles of Fluorescence Spectroscopy**. Boston, MA: Springer US, 2006. p. 353–382.

[5] VALEUR, B. Fluorescence Polarization. Emission Anisotropy. Em: **Molecular Fluorescence**. [s.l.] Wiley, 2001. p. 125–154.

[6] ZHMUD, B. Viscosity Blending Equations. **Lube**, v. 121, p. 24, dez. 2014.

[7] SHEIK-BAHAE, M. et al. Sensitive Measurement of Optical Nonlinearities Using a Single Beam. **IEEE Journal of Quantum Electronics**, v. 26, n. 4, 1990.

[8] GARCIA, R. DE Q. **Ultrafast pump-probe platform for broadband and polarization-resolved characterization of materials: case study of octupolar push-pull azobenzenes**. São Carlos: Universidade de São Paulo, 31 ago. 2023.

[9] SNELLENBURG, J. J. et al. Glotaran: A Java-Based Graphical User Interface for the R Package TIMP. **Journal of Statistical Software**, v. 49, n. 3, 2012.
